# Circ-ZFR Promotes Progression of Bladder Cancer by Upregulating WNT5A *Via* Sponging miR-545 and miR-1270

**DOI:** 10.3389/fonc.2020.596623

**Published:** 2021-04-13

**Authors:** Liping Luo, Pingping Miao, Yao Ming, Jie Tao, Hongchun Shen

**Affiliations:** ^1^Department of Nephrology, The Affiliated Traditional Chinese Medicine Hospital of Southwest Medical University, Luzhou, China; ^2^Department of Nephrology, The Traditional Chinese Medicine Hospital of Luzhou City, Luzhou, China; ^3^College of Integrated Chinese and Western Medicine, Southwest Medical University, Luzhou, China

**Keywords:** circZFR, bladder cancer, miR-545, miR-1270, WNT5A

## Abstract

**Background:**

Bladder cancer is one of the most common cancers all over the world. CircZFR is a circular RNA and has been implicated in tumor generation and invasion. However, the exact role of circZFR in the development of bladder cancer (BCa) remains unknown. This study aimed to investigate the function of circZFR in BCa, and further to probe into the association between circ-ZFR, miR-545/miR-1270 and WNT5A.

**Methods:**

The expression of circZFR in BCa was quantified by qRT-PCR and was positively correlated with the prognosis of BCa patients. Next, the stable knockdown of circZFR BCa cell lines was established and the resulting capacities of proliferation, migration and invasion were measured. The association of circZFR with miR-1270/miR-545 was predicted by circinteractome prediction, and was confirmed by luciferase assay as well as RNA pull down assay. Furthermore, miRNA inhibitors, WNT5A overexpression and Pearson correlation analysis were used to examine the relationship between circZFR, miR-1270/miR-545 and WNT5A.

**Results:**

The expression of CircZFR was up-regulated both in BCa tissues and in BCa cell lines, and was positively correlated with patient survival rates. Blocking of circZFR’s expression by RNA inhibitors suppressed the proliferation, migration and invasion of BCa cells both *in vitro* and *in vivo*. On the other hand, overexpression of target miRNA supported that circZFR directly interact with miR-545 and miR-1270. Moreover, we demonstrated that circZFR promotes the progression of BCa by upregulating WNT5A’s expression *via* sponging miR-545 and miR-1270.

**Conclusions:**

CircZFR promotes the proliferation, migration and invasion of BCa cells by upregulating WNT5A signaling pathway *via* sponging miR-545 and miR-1270. These results provide new insights into the molecular mechanism of circZFR in BCa progression, and more important, a novel target for BCa clinical treatment.

## Introduction

Bladder cancer (BCa) is a notorious cancer worldwide, leading to more than 430,000 new diagnosed cases (1) and more than 165,000 deaths each year (2). BCa can be divided into two groups depending on its distinct pathological feature: 75% of patients count for low-grade non-muscle-invasive bladder cancer (NMIBC) and 25% of patients have high-grade muscle-invasive bladder cancer (MIBC) (3). Surgery, chemotherapy and radiotherapy are main therapies for patients with local tumors, but when the tumor relapses or metastasizes to other organs, these curative approaches are largely futile. Taken together, it is imperative for oncologists to understand the molecular underpinning of BCa development and progression, and find new biomarkers to improve the diagnosis and treatment of BCa.

Circular RNAs (circRNAs) are a noncoding RNA with a covalently closed loop in cancer genomes (4). circRNA are involved in cellular differentiation and tissue homeostasis (5). The skewed circRNA levels lead to various kinds of deadly cancers. CircRNA-specific computation and high-throughput RNA sequencing are main tools for circRNA research (6). Using these methods, the high expression of circZFR was found in thyroid cancer (7), rectal cancer (8), liver cancer (9, 10), lung cancer (11), kidney cancer (12), where circZFRs affect the occurrence and development of tumors through regulating the cell proliferation, invasion and migration.

By sponging microRNAs or proteins, circRNAs act as a potent inhibitor for miRNA expression (13). This circRNA-miRNA interaction, in turn, regulates cell cycle and transcription, signal transduction and epigenetic modulation, which ultimately regulate the cancer cell proliferation, migration, invasion, and differentiation (14). For instance, downregulation of MiR-545 and MiR-1270 by circRNAs has been implicated in tumor formation and migration, including lung cancer (15), glioma (16), oral squamous cell carcinoma (17), cervical cancer (18) and gastric cancer (19). However, whether the same mechanism through circRNAs sponging miR-545 and miR-1270 is applicable to the bladder cancer has not been investigated.

WNT5A signaling pathway is critical in regulating tumor genesis and cancer cell invasion (20). Both *in vitro* and *in vivo* studies have demonstrated that the WNT5A pathway is a pivotal regulator for multiple cell events such as cell invasion, migration, epithelial-mesenchymal transition and inflammation (20). The expression of WNT5A was increased in BCa cells (21, 22), which is presumably caused by miRNAs-mediated pathways. Considering the reported association between circZFR, WNT5A and miRNA, we hypothesize that circZFRs might be involved in BCa progression by regulating the WNT5A expression in a miRNA-mediated manner. To test the hypothesis, we first analyzed the expression of circZFRs in human BCa samples and BCa cell lines. We found that circZFRs levels was increased in both BCa tissues and cell lines. Next, we elucidated that circZFRs promote the proliferation, migration and invasion of BCa cells by upregulating WNT5A signaling pathway *via* sponging miR-1270 and miR-545. These results provide new insights into the diagnosis and prognosis of BCa through circRNA/miRNA/WNT5A pathway.

## Materials and Methods

### Clinical Tissue Sample and CircRNA Microarrays

BCa tumor and adjacent normal tissues were collected between 2014 and 2018 from patients undergoing surgery at the Department of Neurosurgery, The Affiliated Traditional Chinese Medicine Hospital of Southwest Medical University, Luzhou, Sichuan, China. In total, 60 pairs of tumor and adjacent tissues were quickly extracted and freshly frozen in liquid nitrogen. Tissues were stored at −80°C until RNA extraction. The usage of tissues for the study has been approved by the ethics committee of the The Affiliated Traditional Chinese Medicine Hospital of Southwest Medical University. Before using clinical materials for research purposes, all patients signed the informed consent.

For circRNA analyses, sample preparation and microarray hybridization were performed by Kangchen Bio-tech through Affymetrix Human Genome U133 Plus 2.0 Array (Affymetrix, USA).

#### Cell Culture, Transfection, and Treatment

Human BCa cell lines were obtained from Type Culture Collection of the Chinese Academy of Sciences (Shanghai, China). BCa cells were cultured in DMEM medium (Gibco, USA) supplemented with 10% FBS (Gibco), 100 units/ml penicillin and 100 μg/ml streptomycin (Invitrogen, USA). All cells were cultured at 37°C with 5% CO_2_.

J82 and T24 cells were cultured in 35-mm dishes and transfected with a short interfering RNA targeting control shRNA (sh-NC; 100 nM)/circZFR (sh-circZFR). MiR-1270 inhibitor/control inhibitor, miR-545 inhibitor/control inhibitor, miR-1270 mimics/negative control (NC), miR-545 mimics/negative control (NC) were used to confirm the miRNA function. We used WNT5A-overexpressing plasmid/control plasmid (Shanghai Genepharma Biotech Co., Ltd., Shanghai, China) with Lipofectamine 2000 (Invitrogen, Carlsbad, CA) to transfect cells according to the manufacturer’s instructions.

### Quantitative Real-Time PCR

Total RNA was isolated from the tissues or cells by Trizol reagent (TaKaRa, Japan). The expression of RNA was detected by One-Step SYBR PrimeScript RT-PCR Kit with a Quant Studio 6 Flex Quantitative PCR System (Applied Biosystems, USA). The reverse transcription of miR-545 and miR-1270 were achieved by TaqMan MicroRNA Reverse Transcription kit (Vazyme, China). circZFR forward, 5′-AACCACCACAGATTCACTAT-3′, reverse, 5′-AACCACCACAGATTCACTAT-3′; GAPDH forward, 5′-ATAGCACAGCCTGGATAGCAACGTAC-3′, reverse, 5′-CACCTTCTACAATGAGCTGCGTGTG-3′; miR-545 forward, 5′-ACGGCCATACCACCCTGAAC-3′, reverse, 5′-GGCGGTCTCCCATCCAAGTA-3′; miR-1270 forward, 5′-CTGGAGATATGGAAGAGCT-3′, reverse, 5′- CAGTGCGTGTCGTGGAGT-3′; WNT5A forward, 5′-ATTCTTGGTGGTCGCTAGGT-3′, reverse, 5′-TCCTTGAGAAAGTCCTGCCA-3′

### Cell Viability Assay

To determine the viability of J82 and T24 cells, Cell Counting Kit-8 (CCK-8, Beyotime Institute of Biotechnology) assay was used. Cells were plated in 96-well plates with the density of 5000 cells/well and incubated with normal condition for 24 h to allow cells to adhere and proliferate. Each well was incubated with 90 μl DMEM and 10 μl CCK-8 solution at 37°C for 1 h. Sample absorbance was read by SpectraMax M5 microplate reader (Molecular Devices, San Jose, CA, USA) at 450 nm.

#### Cell Cloning Formation Assay

The transfected J82 and T24 cells were seeded into 35 mm dishes at a density of 400 cells/well and maintained in the normal medium. After three weeks, the cells were fixed with 4% paraformaldehyde and staining with 0.1% crystal violet. All the colonies were imaged and counted by microscope (SZX7, Olympus, Tokyo, Japan).

### Cell Migration and Invasion Assays

To detect cell migration or invasion, transwells (8 μm pore size, Costar), coated with or without matrigel (BD Biosciences, USA), were used. Each upper chamber was seeded 1×10^4^ transfected cells suspended in 200 μl of serum-free medium. As for the bottom chamber, medium containing 10% FBS was added as a chemo-attractant. The cells were incubated at 37°C with 5% CO_2_ for 48 h (invasion assay) or 24 h (migration assay). Cotton swabs were used to remove the cells on the top side of the filter after incubation, and the cells on the lower surface were fixed with 4% paraformaldehyde, stained with 0.1% crystal violet. The stained cells were imaged and counted by microscope.

#### Western Blot

J82 and T24 cells were collected at −80°C after indicate treatments. RIPA lysis buffer (Beyotime Biotechnology, Wuxi, China) added with PMSF (1:100, Beyotime Biotechnology) were used to lyse the cells. BCA assay was used to detect the concentration of protein. Quantified protein was separated by 10% SDS-PAGE gels, and transferred to a PVDF membrane (Millipore, Shanghai, China). After blocking in 5% non-fat milk for 2 h, the membrane was incubated with the primary and secondary antibodies. The target protein band were visualized by the ECL procedure (Sigma) and quantified by ImageJ software (NIH, USA).

### Tumor Xenograft Implantation

Six-to eight-week-old female mice (BALB/C-nu) were purchased from the Cancer Institute of the Chinese Academy of Medical Science. After adapting the environment for a week, the mice into two groups (n = 5). Each group of mice was subcutaneously injected sh-NC or sh-circZFR#1 suspending T24 cells (1 × 10^5^) into the right flank. Tumor nodules were estimated with caliper every 7 days. After 28 days, the mice were sacrificed, and the tumors were isolated and took photos. All animal protocols were complied with the guidelines of the Animal Welfare Act and were approved by the Administrate Panel on Laboratory Animal Care of China Medical University.

#### Luciferase Assay

According to the manufacturer’s protocol, J82 and T24 cells were seeded in a 96-well plate and co-transfected with plasmids containing 3′-UTR of wild or mutant fragments from miRNA mimics and circZFR using Lipofectamine3000 (Invitrogen). After 48 h transfection, renilla luciferase activities were normalized consecutively by using Dual-Lucifer Reporter Assay System (Promega, Massachusetts, USA) according to the manufacturer’s instructions. Each assay was repeated in three independent experiments.

#### Biotin-Coupled Probe Pull Down Assay

This experiment was specifically designed to confirm the junction of circZFR and miRNA, and the oligo probe was taken as control. After washed with 4°C phosphate-buffered saline (PBS), 1 × 10^7^ cells were lysed in 1 ml lysis buffer biotinylated probes. Then the positive control (Input), negative control (Biotin-NC), and biotinylated RNAs (Biotin-circZFR) were added 3 μg per tube and incubated at room temperature for 2 h. Streptavidin Magnetic beads (Life Technologies) were added to the cell lysates to prepare a probe–magnetic bead complex. Then wash the beads with lysis buffer for five times. Finally, the pull-down miRNAs were extracted and detected by RT-PCR assay, and using GAPDH as a control.

### Statistics Analyses

All the analyses were performed with GraphPad Prism 5.0 software (GraphPad Software Inc, San Diego, CA). Data were expressed as mean ± SD and *p* < 0.05 was considered as statistically significant. Student’s t test was used to compare the differences between two groups. Correlations were performed by Pearson’s correlation coefficient analysis. KM-plotter curves were used to perform survival analysis and log-rank test for significance.

## Results

### Circ-ZFR Expression Is Positively Correlated With Prognosis of BCa Patients

**Figure 1:**
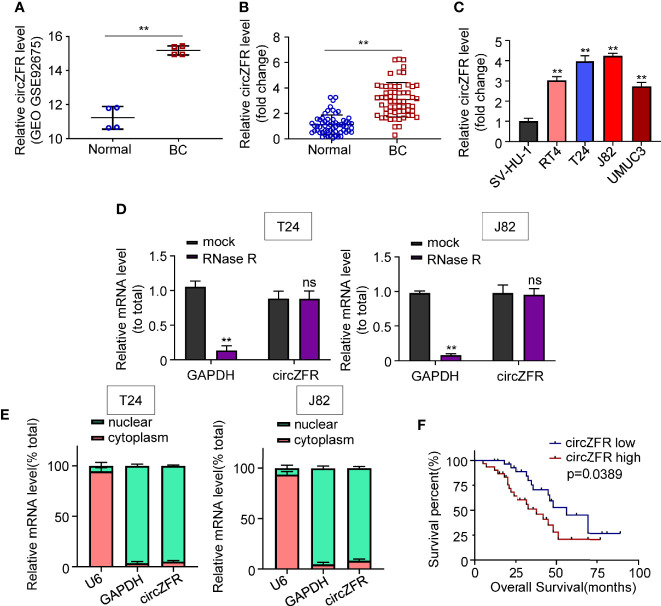
CircZFR is significantly increased in bladder cancer tissues and correlated with prognosis of bladder cancer (BCa) patients. **(A)** Relative expression levels of cirZFR in GSE92675. (**P < 0.01 vs. normal group, *n* = 4) **(B)** CirZFR expression levels in BCa tissues (BC) and adjacent normal tissues (Normal). (**P < 0.01 vs. normal group, *n* = 60) **(C)** Relative cirZFR level of in BCa cell lines and SV-HU-1 cell using. (*P < 0.01 vs. SV-HU-1 cell, *n* = 3) **(D)** mRNA levels of *GAPDH* and circZFR in T24 and J82 cell lines after RNase R treatment. (**P < 0.01 vs. mock group, *n* = 3) **(E)** mRNA levels of u6, GAPDH and circZFR in T24 and J82 cell lines in nuclear and cytoplasm. (*n* = 3) **(F)** Correlation of circZFR expression level with overall survival of BCa patients, analyzed by Kaplan-Meier plotter analysis. (*n* = 60).

To investigate whether circZFR abundance was altered in BCa, four pairs of data were analyzed in GSE92675 dataset. We found that circZFR expression was increased in BCa tumor tissues compared with that in the adjacent normal tissues (**Figure 1A**). Next, circular RNA expression in 60 pairs of tissue was further analyzed and the expression of circZFR was significantly increased in BCa group relative to the control group (**Figure 1B**). Moreover, circZFR levels were positively correlated with TNM stage of BCa and tumor grades of BCa (**Supplementary Figure 1**), which further supports the promotive role of circ-ZFR in tumor development. In addition, circZFR showed a higher expression in RT4, T24, J82, and UMUC3 BCa cell lines relative to a normal urothelial cell line, SV-HU-1 (**Figure 1C**).

To test whether circZFR expression was affected by mRNA or not, we used Ribonuclease R (RNase R) to degrade the mRNA. **Figure 1D** shows that GAPDH expression was decreased after RNase R application compared with mock groups in both T24 and J82 cell lines (**Figure 1D**). By contrast, the circZFR expression was not affected by RNase R in each cell lines (**Figure 1D**). The circZFR’s recalcitrance to RNase R degradation may be due to its localization in the nuclei, which blocks the access to cytosol RNase R. To test this speculation, we then used nuclear separation experiment to locate the RNA expression. As **Figure 1E** shows, circZFR was mainly located in cytoplasm in T24 and J82 cells. This result suggests that circZFR is biochemically resistant to the RNase R degradation.

Additionally, a cohort of 60 BCa patients were divided as a circZFR high expression group and a circZFR low expression group to analyze whether circZFR abundance is correlated with the tumor prognosis. **Table 1** shows that the levels of circZFR were positively correlated with tumor volumes, TNM stages and metastatic lymph node ratios, but not with other clinic pathological characteristics, such as ages, sexes or tumor node metastasis stages. BCa patients with higher circZFR abundances had lower survival rates and vise verse (**Figure 1F**). Taken together, increased circZFR expression leads to a worse prognosis in BCa patients.

**Table 1:** Correlation of circZFR with prognosis of bladder cancer (BCa) patients.

Variable	circZFR expression		*P* value
	Low (n = 30)	High (n = 30)	
Age			
<60	12	15	0.436
≥60	18	15	
Gender			
Male	20	17	0.426
Female	10	13	
Tumor size			
<3 cm	21	6	0.000^*^
≥3 cm	9	24	
Histological grade			
High and middle	5	20	0.020^*^
Low	25	10	
Lymph node metastasis			
Negative	18	9	0.071
Positive	12	21	
Multiplicity			
Single	16	17	0.795
Multiple	14	13	

*P < 0.01,Significant statistical differences between two groups.

### CircZFR Promotes BCa Cell Proliferation, Migration and Invasion

**Figure 2:**
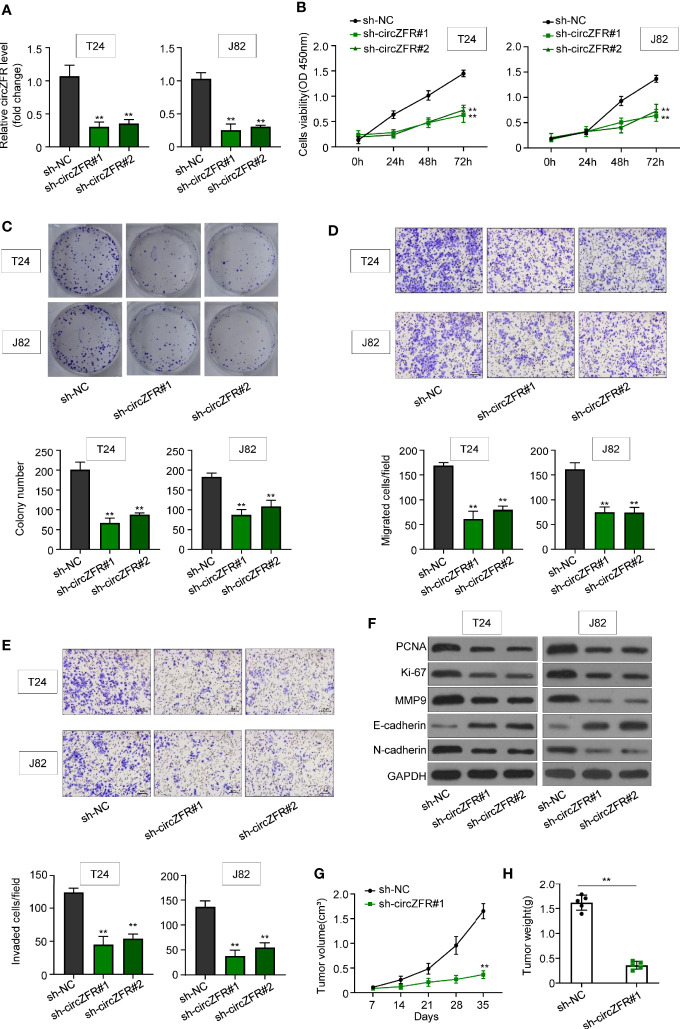
Knockdown of circZFR inhibits bladder cancer (BCa) cells proliferation, migration and invasion. **(A)** Relative circZFR levels in T24 and J82 cells after sh-circZFR#1 or sh-circZFR#2 knockdown. (**P < 0.01 vs. sh-NC group, *n* = 3) **(B)** The effects of circZFR knockdown on BCa cells viability were detected by the CCK8 assay. (**P < 0.01 vs. sh-NC group, *n* = 3) **(C)** Colony formation assay to determine BCa cells colony formation. (**P < 0.01 vs. sh-NC group, n = *4*) **(D, E)** Transwell assay to measure BCa cells migration [**(D)**, without matrigel] and invasion [**(E)**, with matrigel on transwell] capacity. (magnification×100, **P < 0.01 vs. sh-NC group, *n* = 3) **(F)** Protein expression in BCa cells. **(G)** Changes of tumor volume in mice, which were measured per 7 days. (**P < 0.01 vs. sh-NC group, *n* = 5) **(H)** Tumor weight in two groups were measured by electronic scales. (**P < 0.01 vs. sh-NC group, *n* = 5).

To further elucidate the role of circZFR in BCa, circZFR was knocked down in both T24 and J82 cells through transfection with sh-circZFR#1 or sh-circZFR#2. circZFR expression was significantly decreased in knockdown group (**Figure 2A**). Next, the cell proliferation was examined in the established circZFR knockdown cell lines. Knockdown of circZFR in both T24 and J82 cell lines attenuated cell viability (**Figure 2B**). Subsequently, cloning formation assay was used to confirm the proliferation phenotype. circZFR knockdown significantly decreased the colony numbers (**Figure 2C**) and inhibited BCa cell migration and invasion (**Figures 2D, F****)**.

In addition, the abundance of an array of tumor markers, such as PCNA, Ki-67, MMP9, E-cadherin, and N-cardherin were quantified. circZFR knockdown in T24 and J28 significantly decreased the protein abundance of PCNA, Ki-67, MMP9, and N-cardherin, while increasing the E-cardherin protein level (**Figure 2F**). To explore whether circZFR knockdown affects tumor growth *in vivo*, tumor formation of xenograft in nude mice was investigated. Comparing with sh-NC group, circZFR knockdown in BCa cells significantly reduced both tumor volume (**Figure 2G**) and tumor weight (**Figure 2H**) (**Supplementary Figure 2**).

### CircZFR Directly Interacts With miR-545 and miR-1270

**Figure 3:**
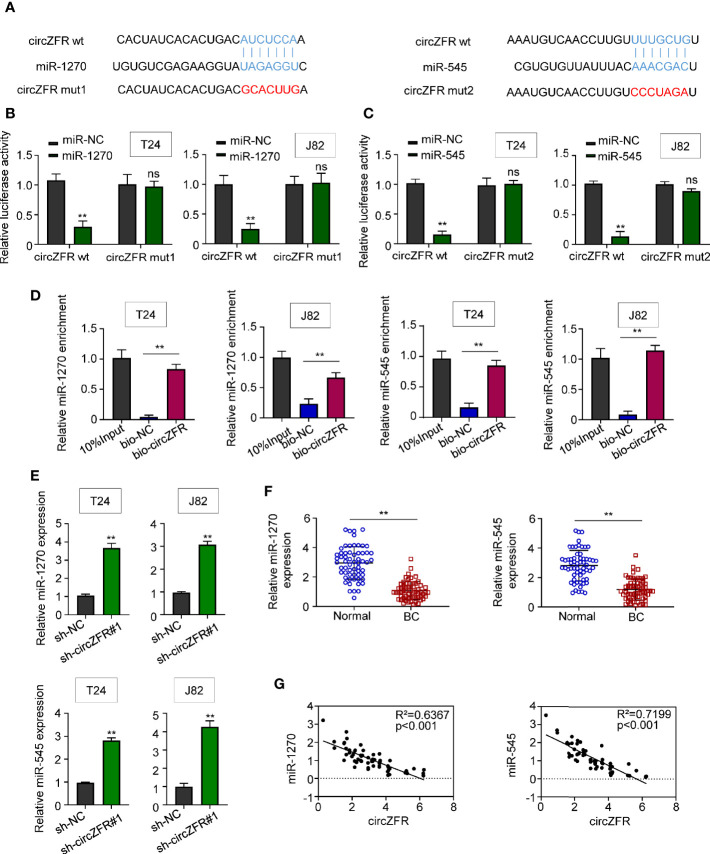
CircZFR functionally targets miR-1270 and miR-545. **(A)** The putative binding sites between circ_0072088 and miR-1270, circ_0072088 and miR-545 were predicted. **(B, C)** Relative luciferase activity in bladder cancer (BCa) cells with or without miR-overexpression. (**P < 0.01 vs. miR-NC group, *n* = 3) **(D)** Quantification of miR-1270 and miR-545 expression by RNA pull-down assay in BCa cells. (*P < 0.01 vs. bio-NC group, *n* = 3) **(E)** MiR-1270 and miR-545 expression in BCa cells. (**P < 0.01 vs. sh-NC group, *n* = 3) **(F)** MiR-1270 and miR-545 expression in human tissues. (**P < 0.01 vs. adjacent normal tissue, *n* = 60) **(G)** Pearson correlation analyses between the expression of circZFR and miR-1270/miR-545.

Using the bioinformatics software, Circinteractome (https://circinteractome.irp.nia.nih.gov/), circ_0072088 was predicted to possess a binding site for miR-1270 and miR-545 (**Figure 3A**). Moreover, overexpression of miR-1207 and miR-545 in both T24 and J82 cells significantly suppressed the luciferase activity in wild type group but not the activity in cells defective in circZFR (**Figures 3B, C****)**. Next, RNA pull-down assay was used to verify the interactions between circZFR and miR-1270/miR-545 or both. In contrast to bio-NC, we found that miR-1270 and miR-545 were highly expressed in bio-circZFR group (**Figure 3D**). Since other circular RNAs can bind to AGO2, we also test whether circ-ZFR could bind to AGO2. The pull-down assay showed that circ-ZFR was enriched in the AGO2 pellet compared with those in the input control (**Supplementary Figure 3**), suggesting circ-ZFR also bind to AGO2 to regulate microRNA levels in tumor cells.

On the other hand, the miR-1270 and miR-545 expressions were quantified in sh-circZFR cells. The expression of both miR-1270 and miR-545 was significantly increased when circZFR was knock-downed in both T24 and J82 cells (**Figure 3E**). The abundances of miR-1270 and miR-545 in 60 pairs of BCa and adjacent tissues were also quantified. Both miRNAs levels were decreased in BCa tissues (**Figure 3F**). Moreover, Pearson correlation analysis shown in **Figure 3G** indicated a strong correlation (*p* < 0.001) between circZFR and miR-1270 (R^2^ = 0.6367), miR-545 (R^2^ = 0.7199). Taken together, these results support a model where circZFR directly interacts with miR-1270 and miR-545 in BCa.

### CircZFR Promotes WNT5A Expression by Sponging miR-545 and miR-1270

**Figure 4:**
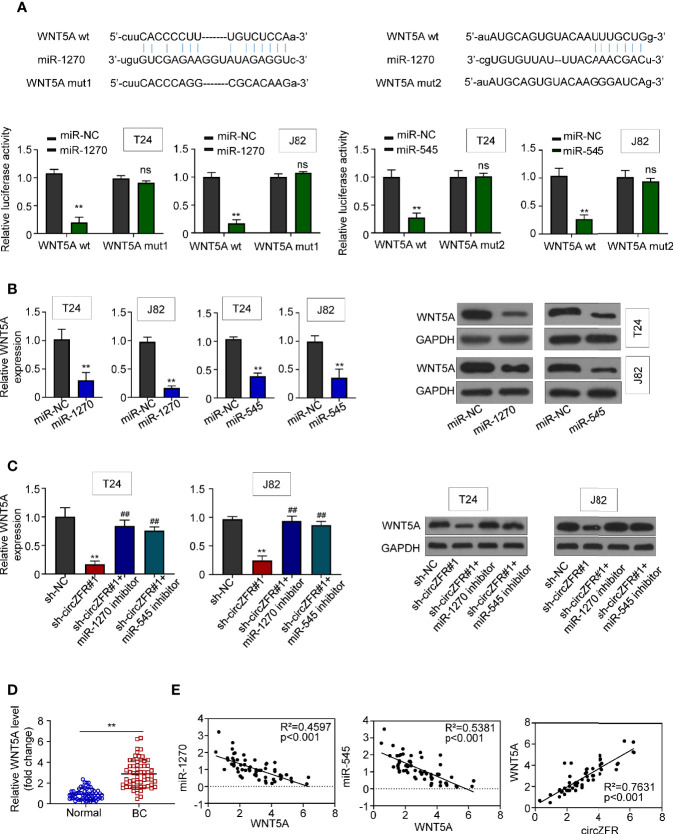
CircZFR modulates WNT5A expression through sponge miR-1270 and miR-545. **(A)** Prediction of putative miR-1270 and miR-545 binding site with WNT5A mRNA and the relative luciferase activity with or without WNT5A mutation. (**P < 0.01 vs. miR-NC, *n* = 3) **(B)** mRNA and protein expression when miR-1270/miR-545 are overexpressed in BCa cells. (**P < 0.01 vs. miR-NC, *n* = 4) **(C)** mRNA and protein expression in circZFR knockdown cells with or without treatment. (**P < 0.01 vs. sh-NC, ^##^*P* < 0.01 vs. sh-circZFR, *n* = 3) **(D)** WNT5A expression levels in BCa tissues (BC) and adjacent normal tissues (Normal). (**P < 0.01 vs. normal group, *n* = 60) **(E)** Pearson correlation analysis between the expression of WNT5A and miR-1270/miR-545, circZFR.

The probability of WNT5A binding with miR-545 and miR-1270 was predicted by Starbase v2.0 (http://starbase.sysu.edu.cn/), and was verified by luciferase reporter assay system. The relative luciferase activity of the miR-1270 and miR-545 group was significantly suppressed as compared with mir-NC group. However, the relative luciferase activity in WNT5A group between miR-NC and miR-1270/545 in BCa cells was not statistically significant (**Figure 4A**). Then, we overexpressed both miR-1270 and miR-545 in T24/J82 cells and measured both mRNA and protein levels of WNT5A. We found that WNT5A, at both mRNA and protein levels, was downregulated in miR-overexpressed group relative to that in miR-NC group (**Figure 4B**).

On the other hand, we wondered whether the expression of WNT5A was increased when the activities of miR-1270 or 545 were inhibited. First, we observed that WNT5A expression was downregulated in sh-circZFR#1 group. However, when we added miR-1270 or miR-545 inhibitors to sh-circZFR#1 cell, we found that WNT5A expression was indistinguishable from that in sh-NC group. Moreover, when either miR-1270 or miR-545 inhibitors was added to T24 or J82 cells, both WNT5A transcript and protein abundances were increased compared with the control groups (**Supplementary Figure 4**). In sum, these results indicated that circZFR regulates WNT5A expression through miRNA pathways (**Figure 4C**).

The BCa tissue contains higher WNT5A levels than normal tissues (**Figure 4D**). Based on the above data, correlation analysis of WNT5A was determined. Moderate correlations were observed between WNT5A and miR-1270 (R^2 =^ 0.4597), miR-545 (R^2 =^ 0.5381). As well, a strong correlation between circZFR and WNT5A was revealed as **Figure 4E**.

### CircZFR Promotes BCa Progression by Regulating miR-545/1270/wnt5a *Via* Wnt/β-Catenin Signaling Pathway

**Figure 5:**
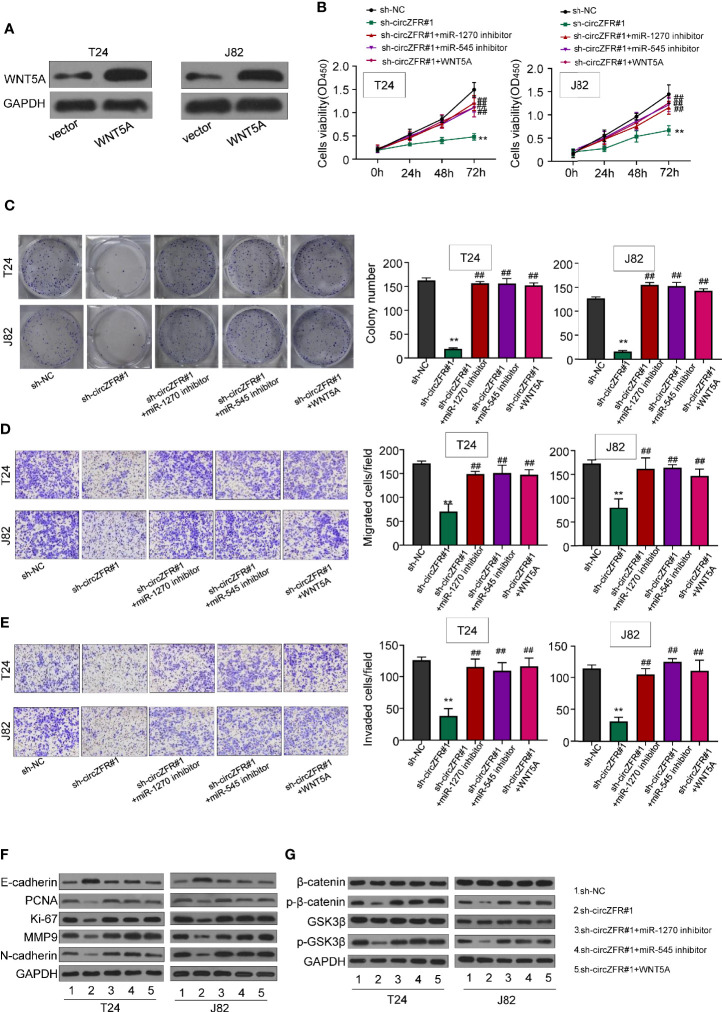
circZFR promotes bladder cancer (BCa) progression by regulating miR-545/1270/wnt5a and *via* activating Wnt/β-catenin signaling pathway. **(A)** Protein expression of T24 and J82 cells. **(B)** Overexpression of miR-1270 inhibitor, miR-545 inhibitor and WNT5A reverses the inhibitory effect of circZFR on cell proliferation revealed by CCK-8 assay (**P < 0.01 vs. sh-NC group, ^##^*P* < 0.01 vs. sh-circZFR group, *n* = 3) **(C)** Colony formation assay to determine BCa cells colony formation. (**P < 0.01 vs. sh-NC group, ^##^*P* < 0.01 vs. sh-circZFR group, *n* = 3) **(D, E)** Transwell assay to measure BCa cells migration [**(D)**, without matrigel] and invasion [**(E)**, with matrigel on transwell] capacity. (magnification×100, **P < 0.01 vs. sh-NC group, ^##^*P* < 0.01 vs. sh-circZFR group, *n* = 3) **(F, G)** Protein expression in T24 and J82 cells.

Having demonstrated the direct interaction between circZFR and miR-1270/545 sponge, and the regulation of WNT5A by miR-1270/miR-545, we reasoned whether circZFR regulates Wnt/β signaling through miR-1270/545 pathway. First, we overexpressed WNT5A in T24 and J82 cell lines (**Figure 5A**). Using CCK8 assay, and we then tested cell viability and found that both application of miR-1270/miR-545 inhibitors and WNT5A overexpression significantly increase the tumor progression in sh-circZFR BCa cells (**Figure 5B**). Furthermore, the colony formation assay data showed that inhibiting miR-1270/545 or overexpressing WNT5A can partially rescue colony formation caused by circZFR knockdown (**Figure 5C**).

Next, we wondered whether the inhibition of miR-1270/545 or WBT5A overexpression could counteract with circZFR knockdown. When circZFR was knocked down, the efficiency of tumor cell migration and invasion were decreased (**Figures 5D, E****)**. However, after the application of miR-1270/545 inhibitors or WNT5A overexpression, the migration and invasion capacity of BCa cells was almost normal compared with that of sh-circZFR group (**Figures 5D, E****)**. Finally, we tested the expression levels of salient signaling components in Wnt/β-Catenin pathway. circZFR knockdown in T24 and J28 significantly decreased PCNA, Ki-67, MMP9 and N-cardherin protein expression (**Figure 5F**), while application of miR-1270/545 inhibitors or WNT5A overexpression partially rescued the reduction (**Figure 5F**). Likewise, in circZFR knockdown cells, the expression of p-β-catenin and p-GSK3β were decreased. miR-1270/545 inhibitors application or WNT5A overexpression also led to a normal p-β-catenin and p-GSK3β protein level (**Figure 5G**).

## Discussion

Considering the high morbidity and mortality of bladder cancer, early diagnosis and efficacious treatment are highly beneficial. This study demonstrated for the first time that circZFR plays an important role in BCa progression. CircZFR and WNT5A were highly expressed in BCa, whereas miR-1270 and miR-545 were sparsely expressed as compared with those in normal tissues. Our results describe the role of circZFR in regulating WNT5A pathway *via* the sponging miRNAs in BCa.

CircZFR is highly expressed in tumor tissues including non-small lung cell cancer (NSCLC) (11), gastric cancer (23), liver cancer (9, 10), colorectal cancer (8), renal carcinoma (12) and thyroid Cancer (7). CircZFR is also highly expressed in 104 pairs of BCa tissues (24). In this study, the elevated expression of circZFR is not only detected in BCa tissues, but also found in BCa cell lines. By acting as a miR-101-3p sponge, circZFR can accelerates NSCLC progression *via* enhancing CUL4B expression and regulating the cancer cell proliferation, invasion and migration mechanism (11). In gastric cancer, circZFR modulates PTEN expression by sponging miR-130a/miR-107 (23). Here, we demonstrated that circZFR directly interacts with miR-545 and miR-1270. Through sponging miR-545/miR-1270, circZFR promotes WNT5A pathway and ultimately accelerates BCa cell proliferation, migration and invasion. We also used the co-transfection and “knockdown + inhibitor” to verify the interaction between miR-545/miR-1270 and WNT5A. Our work supports a model in which the elevated expression of circZFR upregulates WNT5A expression by directly inhibiting miRNA pathways in BCa cells.

The WNT family is an important regulator for cell development and stemness (25). WNT5A, was found to promote cancer cell proliferation and enhance cell migration in nasopharyngeal cancer (26). Previous studies found that WNT/β-catenin signaling stimulate cell invasion and metastasis *via* ARF6 in melanoma cells (27). In bladder cancer, WNT5A regulates BCa cell invasion and migration, which is suppressed by SOX4 (28). Recently, it has been reported that WNT5A expression was partially affected by microRNA. For instance, in ovarian cancer, miR-365 has been found to regulate SKOV3 cells proliferation, colony formation, migration, and invasion through targeting and inhibiting the expression of WNT5A (29). By targeting several pathways of cell migration or proliferation, miR-1270 and miR-545 were known as crucial molecules in multiple cancers (30, 31). After confirming the important role of circZFR in BCa, we further demonstrated that miR-1270/miR-545 were able to bind with circZFR by bioinformatics analysis and biotin-coupled probe pull-down assay. Further correlation analysis and luciferase assay also verified that miR-1270/miR-545 directly target WNT5A and inhibit tumor progression in BCa. Moreover, miRNA inhibitor treatment and WNT5A overexpression reversed the phenotype of circZFR knockdown. Based on the results above, a novel regulatory axis of circZFR-sponging miR-1270/miR-545-WNT5A in BCa is presented. In this model, circ-ZFR play a central role regulate the tumor development. Voluminous reports have confirmed the upregulation of circ-ZFR in various cancers. Yet, the molecular mechanism of this upregulation has not been studied. It has been reported that aberrant chromosome translocation in tumors generates the so-called oncogenic fusion protein that would facilitate the production of circular RNA, contributing to the tumorigenesis (32). This could be the major triggers for circ-ZFR upregulation in tumor cells. This hypothesis needs to be tested in the following work.

BCa can be divided into two groups, NMIBC and MIBC. In the study, we did not distinguish patients based on this criterion. In the future, it would be worthwhile to check whether circZFR is differentially expressed in NMIBC and MIBC groups. In addition, considering the pivotal role of circular RNA in BCa progression, it is imperative and beneficial to find specific circZFR inhibitors for BCa clinical treatment.

## Conclusion

In summary, the expression of circZFR is increased in BCa, which is correlated with poor prognosis of BCa patients. Moreover, knockdown of circZFR inhibits cell proliferation, migration and invasion of BCa cells. Furthermore, we provide evidence to explicitly support an interactive matrix involving circZFR/sponging miR-545 and miR-1270/WNT5A in BCa. This network serves as a potential biomarker and an alternative strategy for BCa treatment.

## Data Availability Statement

The original contributions presented in the study are included in the article/**Supplementary Material**. Further inquiries can be directed to the corresponding author.

## Author Contributions

LL and HS conceived the project and wrote the manuscript. LL and PM performed most of the experiments. YM and JT cultured the cells and ran Q-PCR. LL, PM, and HS analyzed the data. LL and HS supervised the project. All authors contributed to the article and approved the submitted version.

## Conflict of Interest

The authors declare that the research was conducted in the absence of any commercial or financial relationships that could be construed as a potential conflict of interest.

## Supplementary Material

Supplementary Figure 1 CircZFR is significantly increased in advanced stages of and high grade of BCa. **(A)** Relative expression levels of cirZFR in stage III/IV tumors (*n* = 31) relative to stage I/II tumors (*n* = 29). (**P* < 0.01) **(B)** Relative expression levels of cirZFR in high grade tumors (*n* = 27) relative to low grade tumors (*n* = 33). (**P* < 0.01)

Supplementary Figure 2 Representative pictures of tumor xenograft implantation in circ-ZFR know down group and control group.

Supplementary Figure 3 Circ-ZFR binds to Ago2. **(A)** circZFR was enriched in Ago2 pellet in T24 cells. **(B)** circZFR was enriched in Ago2 pellet in J82 cells.

Supplementary Figure 4 WNT5A transcripts and protein levels are increased when either miR-1270 or miR-545 is inhibited.**(A)** Quantification of *WNT5A* transcripts by PCR when either miR-1270 or miR-545 is inhibited in T24 and J82 cells. **(B)** Measurement of WNT5A protein levels by western blot when either miR-1270 or miR-545 is inhibited in T24 and J82 cells.

## References

[1] Antoni S Ferlay J Soerjomataram I Znaor A Jemal A Bray F. Bladder Cancer Incidence and Mortality: A Global Overview and Recent Trends. Eur Urol (2017) 71(1):96–108. 10.1016/j.eururo.2016.06.010 27370177

[2] Alifrangis C McGovern U Freeman A Powles T Linch M. Molecular and histopathology directed therapy for advanced bladder cancer. Nat Rev Urol (2019) 16(8):465–83. 10.1038/s41585-019-0208-0 31289379

[3] Kamat AM Hahn NM Efstathiou JA Lerner SP Malmström PU Choi W. Bladder cancer. Lancet (London England) (2016) 388(10061):2796–810. 10.1016/S0140-6736(16)30512-8 27345655

[4] Kristensen LS Hansen TB Venø MT Kjems J. Circular RNAs in cancer: opportunities and challenges in the field. Oncogene (2018) 37(5):555–65. 10.1038/onc.2017.361 PMC5799710 28991235

[5] Salzman J. Circular RNA Expression: Its Potential Regulation and Function. Trends Genet (2016) 32(5):309–16. 10.1016/j.tig.2016.03.002 PMC4948998 27050930

[6] Hsiao KY Sun HS Tsai SJ. Circular RNA - New member of noncoding RNA with novel functions. Exp Biol Med (Maywood NJ) (2017) 242(11):1136–41. 10.1177/1535370217708978 PMC5478007 28485684

[7] Wei H Pan L Tao D Li R. Circular RNA circZFR contributes to papillary thyroid cancer cell proliferation and invasion by sponging miR-1261 and facilitating C8orf4 expression. Biochem Biophys Res Commun (2018) 503(1):56–61. 10.1016/j.bbrc.2018.05.174 29842886

[8] Bian L Zhi X Ma L Zhang J Chen P Sun S. Hsa_circRNA_103809 regulated the cell proliferation and migration in colorectal cancer via miR-532-3p / FOXO4 axis. Biochem Biophys Res Commun (2018) 505(2):346–52. 10.1016/j.bbrc.2018.09.073 30249393

[9] Tan A Li Q Chen L. CircZFR promotes hepatocellular carcinoma progression through regulating miR-3619-5p/CTNNB1 axis and activating Wnt/β-catenin pathway. Arch Biochem Biophys (2019) 661:196–202. 10.1016/j.abb.2018.11.020 30468709

[10] Yang X Liu L Zou H Zheng YW Wang KP. circZFR promotes cell proliferation and migration by regulating miR-511/AKT1 axis in hepatocellular carcinoma. Dig Liver Dis (2019) 51(10):1446–55. 10.1016/j.dld.2019.04.012 31147216

[11] Zhang H Wang X Hu B Zhang F Wei H Li L. Circular RNA ZFR accelerates non-small cell lung cancer progression by acting as a miR-101-3p sponge to enhance CUL4B expression. Artif Cells Nanomed Biotechnol (2019) 47(1):3410–6. 10.1080/21691401.2019.1652623 31407591

[12] Wang M Gao Y Liu J. Silencing circZFR inhibits the proliferation, migration and invasion of human renal carcinoma cells by regulating miR-206. Onco Targets Ther (2019) 12:7537–50. 10.2147/OTT.S215012 PMC6750881 31571906

[13] Kristensen LS Andersen MS Stagsted LVW Ebbesen KK Hansen TB. The biogenesis, biology and characterization of circular RNAs. Nat Rev Genet (2019) 20: (11):675–91. 10.1038/s41576-019-0158-7 31395983

[14] Panda AC. Circular RNAs Act as miRNA Sponges. Adv Exp Med Biol (2018) 1087:67–79. 10.1007/978-981-13-1426-1_6 30259358

[15] Cui J Pan G He Q Yin L Guo R Bi H. MicroRNA-545 targets ZEB2 to inhibit the development of non-small cell lung cancer by inactivating Wnt/β-catenin pathway. Oncol Lett (2019) 18(3):2931–8. 10.3892/ol.2019.10619 PMC6676444 31452774

[16] Zhang X Yang H Zhao L Li G Duan Y. Circular RNA PRKCI promotes glioma cell progression by inhibiting microRNA-545. Cell Death Dis (2019) 10(8):616. 10.1038/s41419-019-1863-z 31409777 PMC6692337

[17] Yuan G Wu H Du Y He F. Tumor suppressor role of microRNA-545 in oral squamous cell carcinoma. Oncol Lett (2019) 17(2):2063–8. 10.3892/ol.2018.9820 PMC6341794 30675273

[18] Hu C Wang Y Li A Zhang J Xue F Zhu L. Overexpressed circ_0067934 acts as an oncogene to facilitate cervical cancer progression via the miR-545/EIF3C axis. Cell Physiol (2019) 234: (6):9225–32. 10.1002/jcp.27601 30362562

[19] Ma M Zhao J Wu Q Xiao K Li S Zhu H. MiRNA-545 negatively regulates the oncogenic activity of EMS1 in gastric cancer. Cancer Med (2018) 7(6):2452–62. 10.1002/cam4.1520 PMC6010719 29733519

[20] Asem MS Buechler S Wates RB Miller DL Stack MS. Wnt5a Signaling in Cancer. Cancers (2016) 8(9):79. 10.3390/cancers8090079 PMC5040981 27571105

[21] Cao J Wang Q Wu G Li S Wang Q. miR-129-5p inhibits gemcitabine resistance and promotes cell apoptosis of bladder cancer cells by targeting Wnt5a. Int Urol Nephrol (2018) 50(10):1811–9. 10.1007/s11255-018-1959-x 30117016

[22] Chen X Jia C Jia C Jin X Gu X. MicroRNA-374a Inhibits Aggressive Tumor Biological Behavior in Bladder Carcinoma by Suppressing Wnt/β-Catenin Signaling. Cell Physiol Biochem (2018) 48(2):815–26. 10.1159/000491911 30032142

[23] Liu T Liu S Xu Y Shu R Wang F Chen C. Circular RNA-ZFR Inhibited Cell Proliferation and Promoted Apoptosis in Gastric Cancer by Sponging miR-130a/miR-107 and Modulating PTEN. Cancer Res Treat (2018) 50(4):1396–417. 10.4143/crt.2017.537 PMC6192924 29361817

[24] Zhang WY Liu QH Wang TJ Zhao J Cheng XH Wang JS. CircZFR serves as a prognostic marker to promote bladder cancer progression by regulating miR-377/ZEB2 signaling. Biosci Rep (2019) 39(12):BSR20192779. 10.1042/BSR20192779 PMC6893170 31746333

[25] Zhan T Rindtorff N Boutros M. Wnt signaling in cancer. Oncogene (2017) 36: (11):1461–73. 10.1038/onc.2016.304 PMC5357762 27617575

[26] Qin L Yin YT Zheng FJ Peng LX Yang CF Bao YN. WNT5A promotes stemness characteristics in nasopharyngeal carcinoma cells leading to metastasis and tumorigenesis. Oncotarget (2015) 6(12):10239–52. 10.18632/oncotarget.3518 PMC4496352 25823923

[27] Grossmann AH Yoo JH Clancy J Sorensen LK Sedgwick A Tong Z. The small GTPase ARF6 stimulates β-catenin transcriptional activity during WNT5A-mediated melanoma invasion and metastasis. Sci Signaling (2013) 6(265):ra14. 10.1126/scisignal.2003398 PMC3961043 23462101

[28] Moran JD Kim HH Li Z Moreno CS. SOX4 regulates invasion of bladder cancer cells via repression of WNT5a. Int J Oncol (2019) 55(2):359–70. 10.3892/ijo.2019.4832 PMC6615919 31268162

[29] Wang Y Xu C Wang Y Zhang X. MicroRNA-365 inhibits ovarian cancer progression by targeting Wnt5a. Am J Cancer Res (2017) 7(5):1096–106. PMC5446477 28560060

[30] Yuan W Zhou R Wang J Han J Yang X Yu H. Circular RNA Cdr1as sensitizes bladder cancer to cisplatin by upregulating APAF1 expression through miR-1270 inhibition. Mol Oncol (2019) 13(7):1559–76. 10.1002/1878-0261.12523 PMC6599840 31131537

[31] Changjun L Feizhou H Dezhen P Zhao L Xianhai M. MiR-545-3p/MT1M axis regulates cell proliferation, invasion and migration in hepatocellular carcinoma. Biomed Pharmacother (2018) 108:347–54. 10.1016/j.biopha.2018.09.009 30227328

[32] Guarnerio J Bezzi M Jeong JC Paffenholz SV Berry K Naldini MM. Oncogenic ole of fusion-circRNAs derived from cancer-associated chromosomal translocations. Cell (2016) 165:289–302. 10.1016/j.cell.2016.03.020 27040497

