# Supplementary figures and images for: Circ-ZFR Promotes Progression of Bladder Cancer by Upregulating WNT5A Via Sponging miR-545 and miR-1270
Source: Front Oncol. 2021 Apr 13;10:596623. doi: 10.3389/fonc.2020.596623 (PMC8076638; doi:10.3389/fonc.2020.596623)

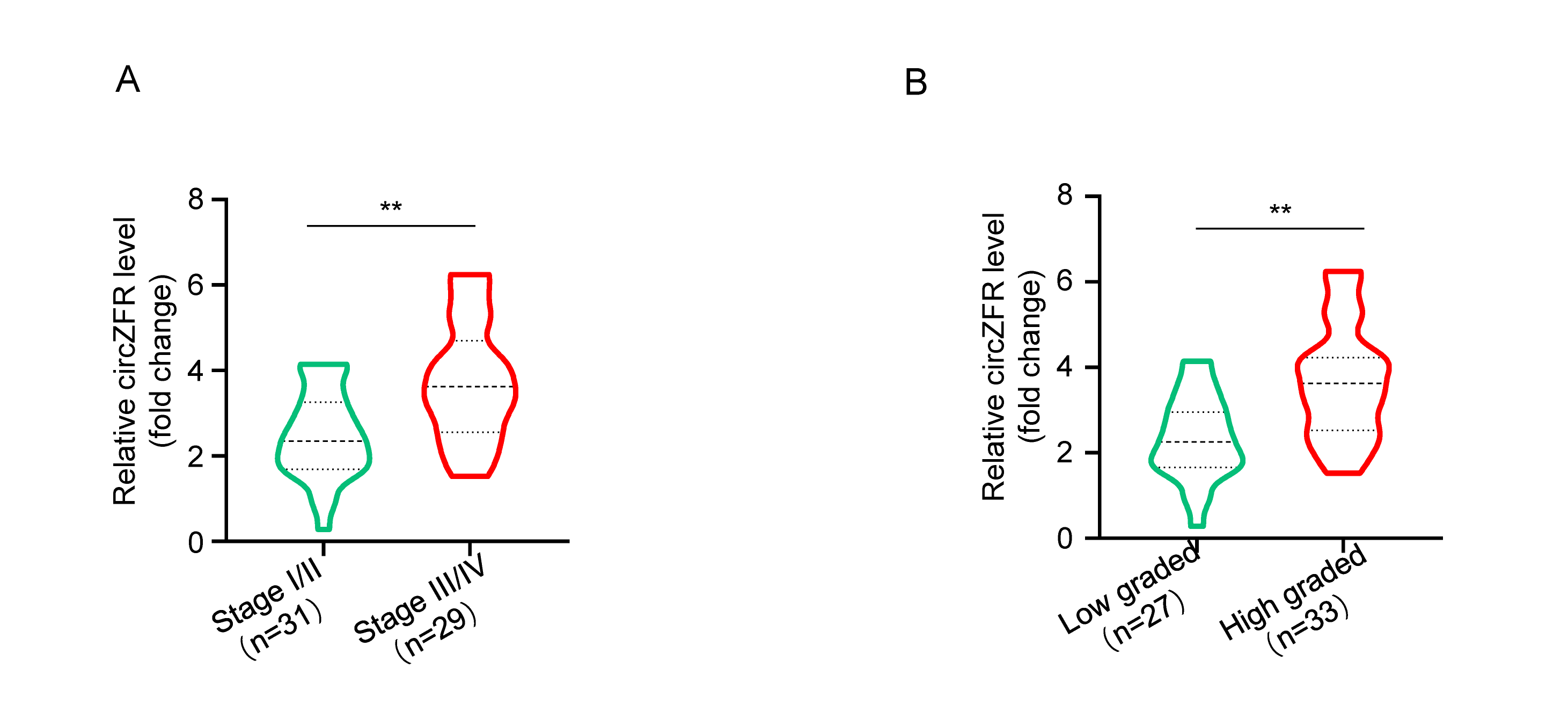

Supplement: Supplementary Figure 1 — CircZFR is significantly increased in advanced stages of and high grade of BCa. (A) Relative expression levels of cirZFR in stage III/IV tumors (n = 31) relative to stage I/II tumors (n = 29). (*P < 0.01) (B) Relative expression levels of cirZFR in high grade tumors (n = 27) relative to low grade tumors (n = 33). (*P < 0.01) [file Image_1.tif]

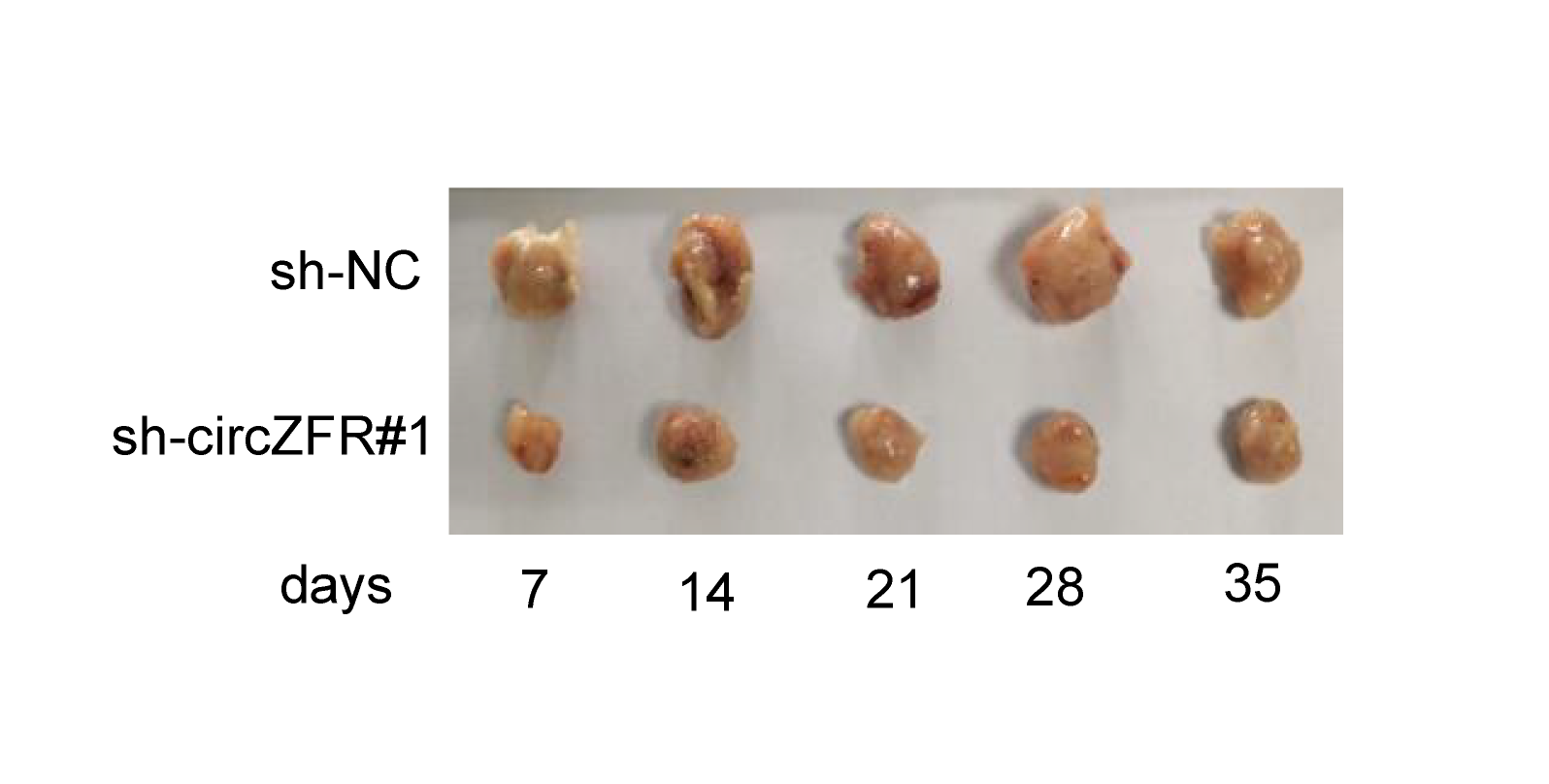

Supplement: Supplementary Figure 2 — Representative pictures of tumor xenograft implantation in circ-ZFR know down group and control group. [file Image_2.tif]

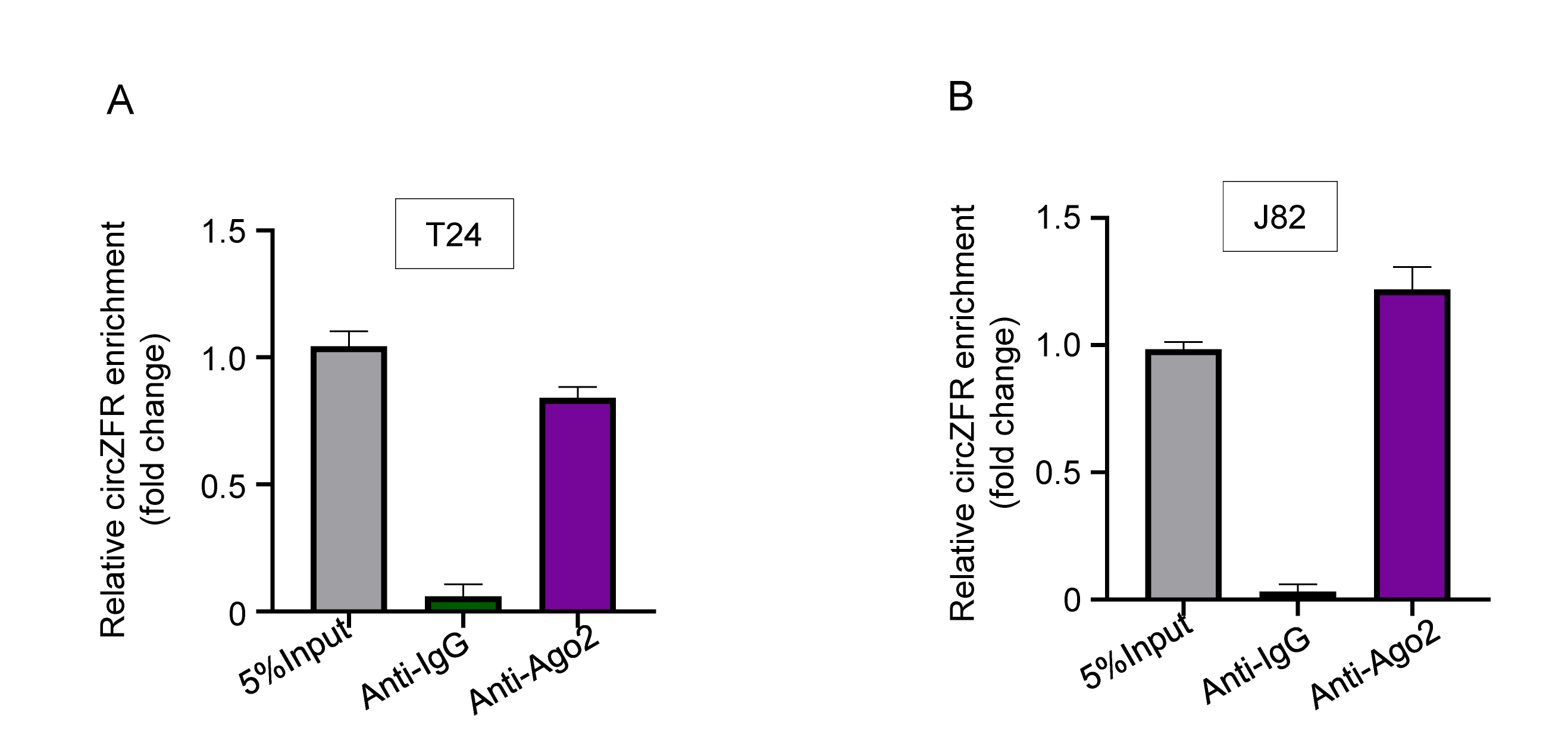

Supplement: Supplementary Figure 3 — Circ-ZFR binds to Ago2. (A) circZFR was enriched in Ago2 pellet in T24 cells. (B) circZFR was enriched in Ago2 pellet in J82 cells. [file Image_3.tif]

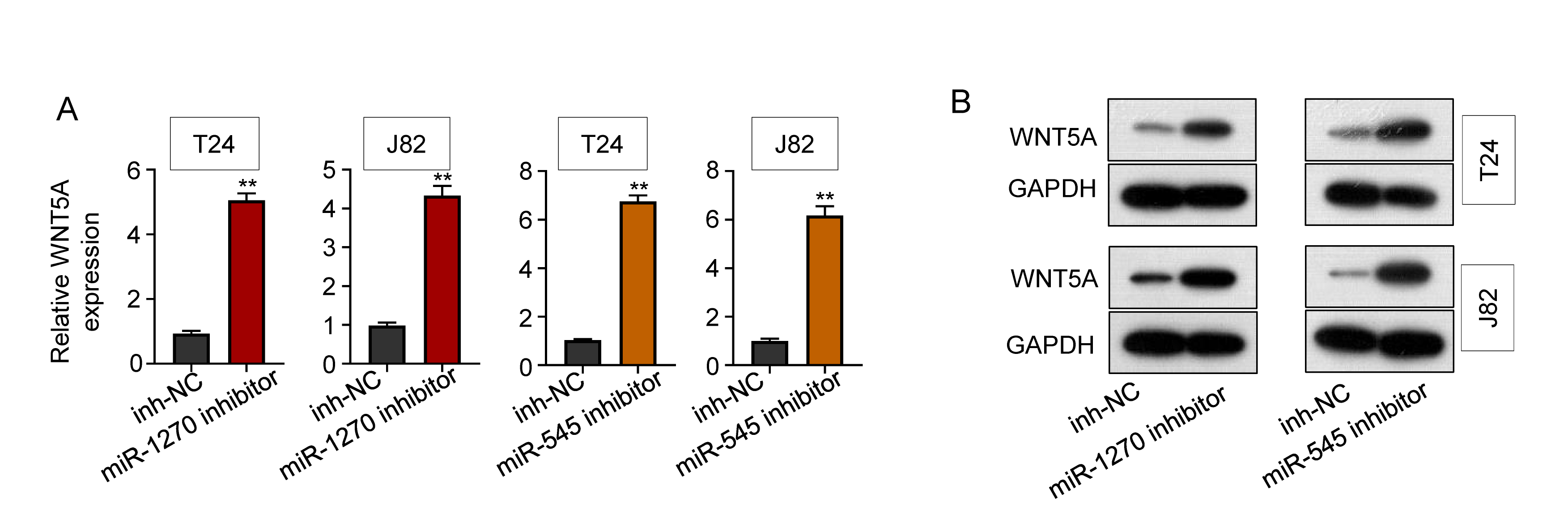

Supplement: Supplementary Figure 4 — WNT5A transcripts and protein levels are increased when either miR-1270 or miR-545 is inhibited.(A) Quantification of WNT5A transcripts by PCR when either miR-1270 or miR-545 is inhibited in T24 and J82 cells. (B) Measurement of WNT5A protein levels by western blot when either miR-1270 or miR-545 is inhibited in T24 and J82 cells. [file Image_4.tif]
